# Are Proselfs More Deceptive and Hypocritical? Social Image Concerns in Appearing Fair

**DOI:** 10.3389/fpsyg.2018.02268

**Published:** 2018-11-21

**Authors:** Honghong Tang, Shun Wang, Zilu Liang, Walter Sinnott-Armstrong, Song Su, Chao Liu

**Affiliations:** ^1^Business School, Beijing Normal University, Beijing, China; ^2^State Key Laboratory of Cognitive Neuroscience and Learning, IDG/McGovern Institute for Brain Research, Beijing Normal University, Beijing, China; ^3^Beijing Key Laboratory of Brain Imaging and Connectomics, Beijing Normal University, Beijing, China; ^4^Philosophy Department and Kenan Institute for Ethics, Duke University, Durham, NC, United States; ^5^Center for Collaboration and Innovation in Brain and Learning Sciences, Beijing Normal University, Beijing, China

**Keywords:** social value orientations, social image concerns, deception, moral hypocrisy, hypocritical fairness, social evaluation

## Abstract

Deception varies across individuals and social contexts. The present research explored how individual difference measured by social value orientations, and situations, affect deception in moral hypocrisy. In two experiments, participants made allocations between themselves and recipients with an opportunity to deceive recipients where recipients cannot reject their allocations. Experiment 1 demonstrated that proselfs were more deceptive and hypocritical than prosocials by lying to be apparently fair, especially when deception was unrevealed. Experiment 2 showed that proselfs were more concerned about social image in deception in moral hypocrisy than prosocials were. They decreased apparent fairness when deception was revealed and evaluated by a third-party reviewer and increased it when deception was evaluated but unrevealed. These results show that prosocials and proselfs differed in pursuing deception and moral hypocrisy social goals and provide implications for decreasing deception and moral hypocrisy.

## Introduction

Although deception is common in daily life, it varies across individuals and social contexts. For example, about 30% people preferred to tell the truth rather than lying, when their lies only benefited themselves but did not affect others’ payoffs (López-Pérez and Spiegelman, 2013). When lies benefit liars but cost others, about 19% people never lie and 10% people lie in more than 60% trials (Tang et al., 2016, 2018). The percentage of liars ranged from 17% to 52% when the benefits decreases and the cost increases (Gneezy, 2005). Specifically, those who were more concerned about social goals were less likely to lie (Cappelen et al., 2013). These variances have attracted significant attention but few studies have been conducted to directly test individual difference in deception and whether and how it is associated with social preference.

Additionally, people not only lie for material benefits, but also lie for non-material goals. They lie to be wiser in achievements or knowledge, to be more attractive in online dating profiles (DePaulo et al., 1996; Toma et al., 2008), and to *appear* fairer to avoid cost of being *truly* fair (C Daniel Batson et al., 1999).Specifically, the phenomenon that appearing moral rather than being truly so has been defined as moral hypocrisy, in which people pursue self-interest by masking unfair behaviors by lying. Most extant studies focused on how material-benefited deception is changed in different social situations (Gneezy, 2005; Jenkins et al., 2016), whereas little is known about how nonmaterial-motivated deception (i.e., moral hypocrisy) is affected by situations.

In this paper, we examined whether individuals with different social values behaved differently when they had opportunities to be morally hypocritical by lying, and whether they changed moral hypocrisy deception when they were confronted with social image concerns in situations. To do so, we measured individuals’ social value orientations and how they behaved in a dictator game when they knew that they could appear fair to their partners by lying. Across two experiments, we manipulated (a) that their hypocritical fairness would be revealed (or not) to their partners and (b) that their hypocritical fairness would be revealed (or not) and evaluated (or not) by an anonymous third-party reviewer. We tested predictions that whether individuals with selfish propensities (i.e., proselfs) behaved more deceptively and hypocritically than those with prosocial propensities (i.e., prosocials) did and whether proselfs were more inclined to change moral hypocrisy deception for social image concerns.

### Social Value Orientations and Deception

One measurement can be used to classify individuals with different social preference is social value orientations (SVO) (Kuhlman and Marshello, 1975). It has been widely used to differentiate individuals in evaluating outcomes between themselves and others in distributions (Bogaert et al., 2008; Balliet et al., 2009). People who care about joint outcomes and equity between self and others are categorized as prosocials; those who tend to maximize their own outcomes without regard for others’ are treated as proselfs; those who seek to maximize difference of outcomes between themselves and others are regarded as competitors. Proselfs and competitors have been classified together as proselfs for they share the selfish propensities (Van Lange and Kuhlman, 1994).

According to these characters, prosocials concern fairness and morality more, thus should deceive less for own benefits; contrarily, proselfs would deceive more to purse self-interest (Steinel, 2015; Cui et al., 2018). Empirical studies have provided evidence for proselfs are more likely to deceive than prosocials in the ultimatum bargaining. They showed that when participants played as allocators, more proselfs (81%) sent a deceptive message to their recipients than prosocials (65%) (Koning et al., 2010); when participants played as recipients, percentage of liars in proselfs was increased to 95% but not changed in prosocials (64%). Specifically, proselfs deceived more than prosocials when they were confronted with losses but did not differ from prosocials when they are confronted with gains (Folmer and De Cremer, 2012). These findings indicate that proselfs are more inclined to change deception to adapt to situations.

### Social Image Concerns in Moral Hypocrisy

A possible measurement for how individual difference and situations affect nonmaterial- motivated deception is moral hypocrisy. Research have shown that people show moral hypocrisy by misreporting the results of a coin flip in allocations tasks to assign a desirable job to themselves and a boring one to others, in which they need to perform the assignment by privately flipping the coin (C. Daniel Batson et al., 1997; C Daniel Batson et al., 1999).

Two possible explanations have been proposed for moral hypocrisy: self-deception and impression management (C Daniel Batson et al., 2002; Lönnqvist et al., 2014; Rustichini and Villeval, 2014). The self-deception explanation regards moral hypocrisy as a way to protect one’s self image by not admitting the conflicts between actual behaviors and moral standards. According to this account, people perceive their behaviors as fair, even though they are actually unfair. The impression management explanation assumes that moral hypocrisy enhances one’s positive social image perceived by others. In that case, apparently fair behaviors are motivated to meet public expectations as they are sensitive to evaluation from others (Caviola and Faulmüller, 2014).

Furthermore, moral hypocrisy has been classified into interpersonal and intrapersonal forms based on the existence of public claims (Graham et al., 2015). Interpersonal moral hypocrisy such as moral duplicity or moral deception involves self and other processing. It is essentially interpersonal. Thus, it might be closer to impression management and more sensitive to situational contexts. Whereas intrapersonal moral hypocrisy is caused by the conflicts between moral values and behaviors without public claims, which could be linked with self-deception. Therefore, situations with or without social image concerns might shape moral hypocrisy in different directions, and take different effects on prosocials and proselfs since proselfs are more responsive to situations (Folmer and De Cremer, 2012).

### The Present Research

Given that proselfs deceive more than prosocials for material benefits, we hypothesized that they also show more deception than prosocials in nonmaterial-motivated moral hypocrisy. Further, as proselfs’ deceptive behaviors and interpersonal moral hypocrisy are both situation-specific (Koning et al., 2010; Graham et al., 2015), we hypothesized that proselfs would show greater changes in moral hypocrisy in situations with public claims than prosocials.

We tested our hypotheses with a modified dictator game, in which participants played as proposers and made an allocation of a total amount of money between themselves and recipients. The recipients can only accept allocations, making payoffs of both sides determined by participants. Specifically, only proposers knew the total amount for division before they divided it. They had an opportunity to either tell the true total or misreport it to recipients, providing alternatives for them to be truly fair or apparently fair (i.e., be hypocritical by lying). These alternatives also make it possible to compare participants’ changes of true and hypocritical fairness between different situations. In Experiment 1, we measured participants’ SVO and manipulated situations by telling the proposers that the deception would be revealed or not to recipients (i.e., proposers knew that true or hypocritical fairness with deception would be known by recipients or not) to test how individual difference and situations affect moral hypocrisy. In Experiment 2, we strengthened social image concerns by adding social evaluation from a third party to further examine its effects on moral hypocrisy of prosocials and proselfs.

## Experiment 1

### Method

#### Participants and Design

A 2 (SVO: Prosocial vs. Proself) × 2 (Revelation: R vs. UR) between-subject design was run. One hundred and sixty Chinese participants (117 females and 43 males; mean age = 21.4 years) were recruited from university campus in Beijing and were randomly assigned to R and UR conditions and paid for participation. Four participants did not finish the task, five participants did not know that they could deceive in the task and four participants doubted the recipients might not be real humans. The final analysis was carried out on the remaining 147 valid participants’ data (R: *n* = 74; UR: *N* = 73). The Institutional Review Board of the State Key Laboratory of Cognitive Neuroscience and Learning at Beijing Normal University approved this study.

#### Procedure

**FIGURE 1:**
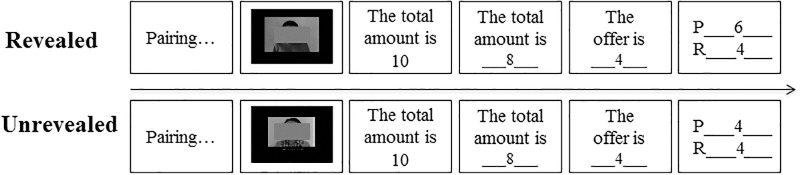
The procedure of Experiment 1. Two sample trials was shown, in which both gains of P (proposer) and R (recipient) based on P’s true total would be revealed (R) or only the gains based on P’s reported total would be revealed and their true totals would not be revealed (UR).

Participants signed consent and were instructed to play as proposers in a modified dictator game with different recipients in 16 trials (photos of programmed recipients were shown). They could either tell recipients the true total (randomly chosen from monetary units: 8, 10, 12, 14) or misreport it while making an allocation between themselves and recipients. Recipients could not reject their allocations, thus the proposers decided payments of both sides. Half of proposers learned that their true totals would be revealed to recipients after the whole experiment (R), and the other half learned that their true totals would be unrevealed to recipients, and recipients would only know the reported total (UR) (Figure 1). Before the formal experiment, participants answered a comprehension quiz, including that whether the recipients would knew their true totals when they made allocation and after the whole experiment, whether the recipients were different in each trial, and whether the recipients could reject their allocations or not.

We recruited participants in three waves. Participants in the first wave (*n* = 30) were required to finish the game firstly, and then completed questionnaires including their feelings about the recipients in the task. After that, we used the six primary SVO slider items to measure social value orientations (Murphy et al., 2011). To make our study comparable with previous studies, we classified participants into prosocials and proselfs based on the angels of SVO. In order to control the distribution of prosocials and proselfs in the R and UR condition, participants in the second and third waves finished the SVO items online before they arrived the lab. In total, 56.8% participants (*n* = 42) in R and 52.1% (*n* = 38) in UR conditions are categorized as prosocials, 43.2% participants (*n* = 32) in R and 47.9% (*n* = 35) in UR conditions are categorized as proselfs.

Finally, participants were debriefed about whether they knew that they could spontaneously misreport their totals and what kind of persons would the recipients be in their opinions, which was used to exclude those participants who did not correctly understand the task and thought the recipients were not real humans. All of them were paid 15 to 25 Yuan (Renminbi: RMB; about $3–$4 at the time of the experiment) depending on their divisions.

### Results and Discussion

#### Deception Rate

**FIGURE 2:**
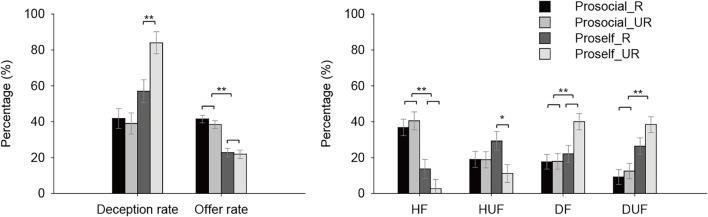
Mean deception rate and offer rate(left), and HF, HUF, DF, and DUF rate of prosocials and proselfs when participants’ true totals would be revealed (R) or not (UR) (right) in Experiment 1. Error bar represents standard errors of the mean (^∗∗^*p* < 0.01, ^∗^*p* < 0.05).

The deception rate was significantly higher than 0 across all conditions (one-sample *t* test, *ts* > 6.71, *ps* < 0.001). A 2 (SVO) × 2 (Revelation) ANOVA analysis found significant main effect of SVO [*F*(1,143) = 26.44, *p* < 0.001, $\eta_{p}^{2}$ = 0.16], Revelation [*F*(1,143) = 4.53, *p* = 0.04, $\eta_{p}^{2}$ = 0.03], interaction of SVO × Revelation [*F*(1,143) = 6.77, *p* = 0.01, $\eta_{p}^{2}$ = 0.05]. Proselfs deceived more than prosocials in both R and UR conditions (*Fs* > 3.23, *ps* < 0.07, $\eta_{\mathrm{ps}}^{2}$ > 0.02); proselfs deceived more in the UR than in R condition [*F*(1,143) = 10.28, *p* = 0.002, $\eta_{p}^{2}$ = 0.07], whereas prosocials did not differ in deception in two conditions [*F*(1,143) = 0.12, *p* = 0.73] (Figure 2). Since the distribution of gender was extremely unequal in this experiment, we did not test the gender difference on deception rate in Experiment 1 but did that in Experiment 2.

#### Offer Rate

The same analysis on offer rate did not find main effect of Revelation, or interaction of SVO × Revelation (*Fs* < 0.62, *ps* > 0.43), which suggest that the participants’ divisions were not affected by Revelation. Main effect of SVO was significant, showing that prosocials gave more to the recipients than proselfs [*F*(1,143) = 58.48, *p* < 0.001, $\eta_{p}^{2}$ = 0.29].

#### Types of Offers

Next, in order to directly compare participants’ hypocritical fairness, true fairness, and true unfairness, we classified proposers’ offers based on whether proposers told the truth (Honest, Deceptive) and whether the reported offer was equal to, less than, or more than 50% of the reported total (Fair, Unfair, Altruistic). This division yielded six different types of offers (participants were told to not report a total higher than the true total): Honest-Fair (HF, true fairness), Honest-Unfair (HUF, true unfairness), Honest-Altruistic (HA, true altruism), Deceptive-Fair (DF, apparent fairness), Deceptive-Unfair (DUF) and Deceptive-Altruistic (DA) (see Table 1). Importantly, both HUF, DF, and DUF led to unfair offers, but DF appeared fairer than HUF and DUF. This feature makes it possible to differentiate participants’ preferences between being truly and apparently fair. Our analysis focused on HF, HUF, DF, and DUF, because percentage of these four types of offers is over 88%.

**Table 1:** Definitions of six types of offers.

Types of offer	Reported Total	Offer
Honest-Fair (HF, true fairness)	= True total	= 50% of true total
Honest-Unfair (HUF, true unfairness)	= True total	< 50% of true total
Honest-Altruistic (HA, true altruism)	= True total	> 50% of true total
Deceptive-Fair (DF, apparent fairness)	< True total	= 50% of reported total
Deceptive-Unfair (DUF)	< True total	< 50% of reported total
Deceptive-Altruistic (DA)	< True total	> 50% of reported total

A mixed 2 (SVO) × 2 (Revelation) × 4 (Types) ANOVA analysis showed significant interaction of SVO × Types, Revelation × Types (*Fs* > 2.76, *ps* < 0.04, $\eta_{p}^{2}$ > 0.02), and three-way interaction of SVO × Revelation × Types [*F*(3,429) = 2.71, *p* = 0.045, $\eta_{p}^{2}$ = 0.02]. Further analysis showed that prosocials made less DF than proselfs in the UR condition [*F*(1,143) = 12.30, *p* = 0.001, $\eta_{p}^{2}$ = 0.08], proselfs decreased HUF [*F*(1,143) = 6.44, *p* = 0.01, $\eta_{p}^{2}$ = 0.04] and increased DF [*F*(1,143) = 7.45, *p* = 0.007, $\eta_{p}^{2}$ = 0.05] in the UR than in R condition.

Experiment 1 showed that proselfs behaved more deceptively and hypocritically than prosocials, especially when their truth would be unrevealed (UR). Specifically, proselfs were more deceptive and hypocritical in UR than in R condition, whereas prosocials were not so sensitive to deception revelation. These findings suggest that proselfs concerned social context more than prosocials. To strengthen the effects of situations and test whether social image differently affects prosocials and proselfs, we added a third-party reviewer of proposers’ behaviors in Experiment 2.

## Experiment 2

In Experiment 2, we added a manipulation that participants’ behaviors would be evaluated by a third-party reviewer which could enhance social image concerns (Nettle et al., 2013). We hypothesized that evaluation by a third party would increase deception and hypocritical fairness when the truth would not be revealed than the truth would be revealed, especially for proselfs.

### Method

#### Participants and Design

A 2 (SVO: Prosocial vs. Proselfs) × 2 (Revelation: R vs. UR) × 2 (Evaluation: Not vs. Eva) between-subject design was used. It results into four conditions for prosocials and proselfs: the true total would be revealed but the offer would not be evaluated (R); the true total would be revealed, and the offer would be evaluated by a third-party reviewer who knows the true and reported totals (R_Eva); the true total would be unrevealed and the offer would not be evaluated (UR); the true total would be unrevealed, but the offer would be evaluated by a third-party reviewer who knows the reported but not the true total (UR_Eva).

Five-hundred and twenty-six Chinese participants finished this study online using the Qualtrics platform in China. Eighty-one participants were excluded for failing to answer the checking questions, resulting in 445 valid participants (304 women and 141 men; mean age = 24.68 years), in which there were 109, 82, 134, and 120 participants in the R, R_Eva, UR, and UR_Eva conditions, respectively.

#### Procedure

Participants played as proposers and allocated a total amount of money (randomly chosen from monetary units: 8, 10, 12, 14) between themselves and eight different recipients, one in each trial. They were told that the recipients could not reject their allocations and did not know the true total amount allocated, and needed to report a total to recipients.

Participants were randomly assigned to four conditions. As in Experiment 1, participants were told that their true totals would be finally revealed (R) or unrevealed to recipients (UR). In R_Eva condition, participants were told that the true total would be presented to the recipients and also that their true totals, reported totals, and offers would be presented to an anonymous third-party reviewer, who would evaluate and rank their offers among all other proposers’ offers. In UR_Eva condition, the recipients would not know the true total, and only the reported total and offers would be evaluated and ranked by an anonymous third-party reviewer, who would not know the true total.

All participants learned the rules and finished a quiz, including whether the recipients could reject the allocation, whether they would know the true totals in all conditions, and whether the third-party reviewer would know the true totals in the R_Eva and UR_Eva conditions. Then they finished the formal experiment. To double check that they actually understand the task, they were required to answer checking questions including whether the recipients would know the true totals in all conditions and whether the third-party reviewer would know the true totals in R_Eva and UR_Eva conditions. Only those who correctly answered these questions were included in the analysis. Next, participants completed the SVO slider items and were paid 6 RMB for participation and a bonus of 4–6 RMB (about $1.5 to $1.8 at the time of the experiment) according to their allocations.

Based on the results in SVO, 53.2 % (*n* = 58 in U condition, 24 men), 63.4% (*n* = 52 in U_Eva condition, 11 men), 60.4% (*n* = 81 in UR condition, 25 men), 54.2% (*n* = 65 in UR_Eva condition, 26 men) participants were classified into prosocials; 46.8% (*n* = 51, 13 men), 36.6% (*n* = 30, 7 men), 39.6% (*n* = 53, 20 men), 45.8% (*n* = 55, 15 men) participants were classified into proselfs, respectively.

### Results and Discussion

#### Deception Rate

**FIGURE 3:**
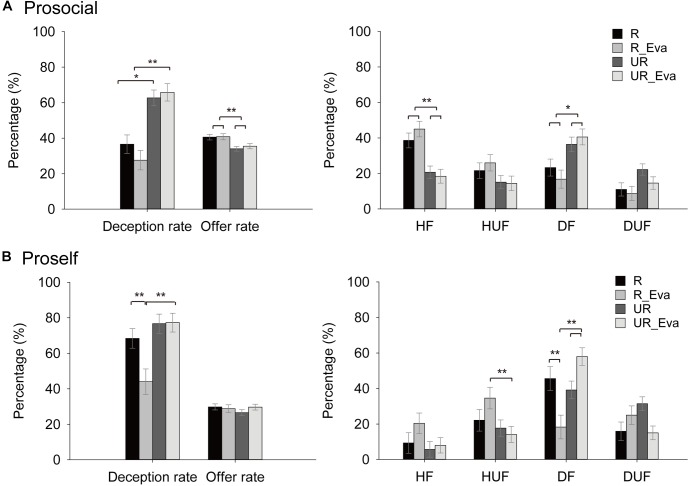
Results in Experiment 2. **(A)** Prosocials’ deception rate, offer rate, and type of offers across conditions, which suggest that prosocials were sensitive to deception revelation rather than evaluation. **(B)** Proselfs’ deception rate, offer rate, and type of offers across conditions, which indicate that proselfs were more sensitive to evaluation, and decreased deception in moral hypocrisy when they faced both deception revelation and evaluation (^∗∗^*p* < 0.01, ^∗^*p* < 0.05).

Results showed that deception rate was higher than 0 across all conditions (one-sample *t* test, *ts* > 6.03, *ps* < 0.001). A 2 (SVO) × 2 (Revelation) × 2 (Evaluation) ANOVA analysis showed significant main effects of SVO (*F*(1,437) = 22.81, *p* < 0.001, $\eta_{p}^{2}$ = 0.05), Revelation (*F*(1,437) = 45.78, *p* < 0.001, $\eta_{p}^{2}$ = 0.095), marginal effect of Evaluation (*F*(1,437) = 3.50, *p* = 0.06, $\eta_{p}^{2}$ = 0.008), and significant interaction of Revelation × Evaluation (*F*(1,437) = 5.53, *p* = 0.019, $\eta_{p}^{2}$ = 0.012) (Figure 3). Prosocials deceived less than proselfs and participants deceived more in the UR than R conditions. The simple effect test of Revelation × Evaluation showed that deception rate was decreased in the R_Eva condition than in the R condition (*F*(1,437) = 7.69, *p* = 0.006, $\eta_{p}^{2}$ = 0.017), which is more significant for proselfs (*F*(1,437) = 6.84, *p* = 0.009, $\eta_{p}^{2}$ = 0.015) than prosocials (*F*(1,437) = 1.42, *p* = 0.23). These findings suggest that participants, especially proselfs were more sensitive to social evaluation. They decreased tendency to behave hypocritically when they knew they would be evaluated by others.

**FIGURE 4:**
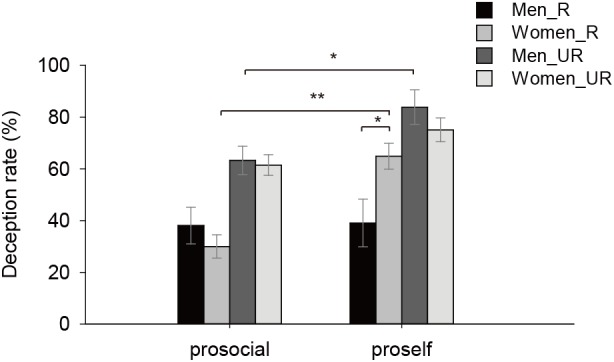
Gender difference in Experiment 2. Proself men deceived less than proself women in the R condition, and deceived more than prosocial men in the UR condition. (^∗∗^*p* < 0.01, ^∗^*p* < 0.05).

We also examined the gender difference in deception rate with a 2 (Gender) × 2 (SVO) × 2 (Revelation) × 2 (Evaluation) ANOVA analysis. Main effect of Gender, two-way and four-way interaction of Gender and other factors were not significant (*Fs* < 2.34, *ps* > 0.13). The interaction of Gender × SVO × Revelation was significant (*F*(1,429) = 5.65, *p* = 0.02, $\eta_{p}^{2}$ = 0.01), then we combined the data in the conditions with and without evaluation to test this interaction. Results showed that proself women deceived more than proself men when their truth would be revealed (*F*(1,429) = 4.53, *p* = 0.03, $\eta_{p}^{2}$ = 0.01). Proself women deceived more than prosocial women in the revealed conditions (*F*(1,429) = 20.90, *p* < 0.001, $\eta_{p}^{2}$ = 0.05), whereas proself men did not differ from prosocial men in these conditions (*F* < 0.007, *p* > 0.93), suggesting that proself men were more sensitive to revelation of truth than proself women (Figure 4).

#### Offer Rate

However, the same analysis on offer rate did not find main effect of Evaluation or interaction of Revelation × Evaluation (*Fs* < 1.26, *ps* > 0.26), which suggests that evaluation did not affect participants’ actual benefits in division. Main effect of Revelation, **SVO,** and interaction of Revelation × SVO were significant (*F*s > 4.18, *ps* < 0.04, $\eta_{\mathrm{ps}}^{2}$ > 0.009), which suggest that prosocials (*F*(1,437) = 15.19, *p* < 0.001, $\eta_{p}^{2}$ = 0.03) increased their actual offers to recipients in the U condition while proselfs did not (*F*(1,437) = 0.38, *p* = 0.54). No gender difference or interaction of gender and other factors were found in the offer rate (*Fs* < 0.64, *ps* > 0.42).

#### Types of Offers

To characterize how participants changed their behaviors, a 2 (Revelation) × 2 (Evaluation) × 2 (SVO) × 4 (Type) mixed ANOVA analysis was run on their different types in presenting offers. The four-way and the Revelation × Evaluation two-way interactions were significant (*F*(3,1311) = 2.74, *p* = 0.04, $\eta_{p}^{2}$ = 0.006; *F*(1,1311) = 4.40, *p* = 0.037, $\eta_{p}^{2}$ = 0.01, respectively) (Figure 3). Then we focused on the effects of Revelation and Evaluation on prosocials and proselfs, respectively in the following analysis.

Prosocials decreased HF (*F*s > 10.7, *ps* < 0.001, $\eta_{\mathrm{ps}}^{2}$ > 0.024) and increased DF (*F*s > 4.34, *ps* < 0.038, $\eta_{\mathrm{ps}}^{2}$ > 0.003) in the UR and UR_Eva conditions compared to R and R_Eva conditions. Their offers were not influenced by Evaluation (*Fs* < 2.57, *ps* > 0.11). In contrast, proselfs decreased HUF and increased DF in the UR_Eva compared to R_Eva (*F*(1,437) = 7.34, *p* = 0.007, $\eta_{p}^{2}$ = 0.017; *F*(1,1311) = 22.67, *p* < 0.001, $\eta_{p}^{2}$ = 0.05, respectively). Specifically, we found that their DF showed an effect as R > R_Eva, and R_Eva < UR < UR_Eva (*F*s > 7.1, *ps* < 0.008, $\eta_{\mathrm{ps}}^{2}$ > 0.016). These findings suggest that prosocials and proselfs responded differently to Revelation and Evaluation. No gender difference was found among these comparisons (*Fs* < 1.81, *ps* > 0.14).

Overall, Experiment 2 replicated the findings of Experiment 1 that that prosocials deceived less than proselfs. It also showed that participants deceived more in UR than in R condition, which is stronger than that in Experiment 1, suggesting that anonymous manipulation strengthens the role of deception revelation in deception and moral hypocrisy. Moreover, the effect of deception revelation on deception in moral hypocrisy was enhanced by adding the evaluation by a third-party reviewer. These results indicated that deception in moral hypocrisy was affected by social image concerns generated by existing of third parties.

Further, prosocials and proselfs behaved differently under the manipulation of deception revelation and evaluation. Prosocials were sensitive to deception revelation but not to evaluation. They decreased their actual offers when their true totals would be revealed by decreasing true fair offers and increasing apparent fair offers. However, proselfs were more sensitive to evaluation, especially when their true totals would be revealed and evaluated by others. They reduced true unfair offers and increased apparent fair offers when they would be evaluated but their deception would be unrevealed, but decreased apparent fair offer when they would be evaluated with deception revelation. These patterns suggests that proselfs are more responsive to social image concerns than prosocials in deception and moral hypocrisy.

## General Discussion

Across two experiments, we found that participants showed great individual difference in hypocritical fairness by deceiving in different situations. Experiment 1 demonstrated that the individual difference could be characterized by SVO, as proselfs deceived more and behaved more hypocritically than prosocials and they are more sensitive to in deception revelation. Experiment 2 provides further evidence that social image concerns take stronger effects on proselfs than prosocials, since existence of a third-party reviewer only led proselfs to decrease apparent fairness when deception was revealed and increase it when deception was not revealed. Taken together, our results suggest that proselfs are more hypocritical than prosocials, especially when they are confronted with social image concerns.

Difference of deceptive behaviors between prosocials and proselfs is considered to be caused by their different social goals (Strombach et al., 2015). According to this conceptual model, both prosocials and proselfs would choose to lie if lying is the most effective way to reach their goals. Thus, prosocials would lie for prosocial motives such as helping others with white lies, which decreased moral conflicts they are confronted with in deception; whereas, proselfs would lie for selfish motives such as gaining more material reward (Sun et al., 2015; Cui et al., 2018). Our results support this model and extend it into moral hypocrisy. One goal of being moral hypocrisy is to avoid cost of being truly moral (C Daniel Batson et al., 1999), which is consistent with proselfs’ selfish motives but contrary to prosocials’ prosocial motives. Thus, proselfs behave more hypocritically than prosocials.

Furthermore, the results that proselfs preferred apparent fairness more than truly unfairness suggest that they behaved hypocritically for nonmaterial goals since the material outcomes for these two types of behaviors would be same. The existence of social evaluation from a hird-party reviewer in Experiment 2 then highlight the importance ofsocial image concerns for proselfs. This finding is consistent with previous studies that show proselfs tended to be fairer and more generous when their behaviors would be perceived by others (Van Dijk et al., 2004). Proselfs mightuse apparent fairness as a strategy to avoid being perceived as unfair partners by others, since people usually use social evaluation to recognize prosocial and antisocial partners (Abdai, 2016), or to avoid punishment andexclusion in the future (Henrich et al., 2010; Gausel and Leach, 2011).

Interestingly, proself men were more sensitive to revelation of truth than proself women. Findings about whether women and men differ in deception are inconsistent in previous studies. For instance, women lie more in non-anonymous conversations than men with expectation of future interactions (Tyler and Feldman, 2006). Men are more likely to lie than women in anonymous and private contexts (Dreber and Johannesson, 2008; Houser et al., 2012). However, some researchers showed that men and women did not differ in frequency of lying but showed difference in the types of lies (DePaulo et al., 1996; Feldman et al., 2002; Childs, 2012). That is, men tend to tell more lies about abilities, personal characteristics, and plans, whereas women lie more about feelings. These findings suggest that men are less responsive to interpersonal processes in deception than women. As it would be more difficult for them in generating lies (Marchewka et al., 2012), they deceive more in contexts without interpersonal cues, but reduce deception in interpersonal contexts. In our study, revelation of truth would expose participants’ hypocritical fairness to recipients, which is closely associated with interpersonal processes, leading proself men to deceive less than proself women.

These findings also provide implications for decreasing deception in moral hypocrisy. As previous studies show, great fraction of people show moral hypocrisy [i.e., 90% in Baston et.al.’s Study 2 (C. Daniel Batson et al., 1997), 100% in Lönnqvist et al.’s Study 1 (Lönnqvist et al., 2014), about 90% in our previous study (Tang et al., 2017)]. However, research about how to decrease it is still at the early stage. Although increasing the concerns of self-image could decrease moral hypocrisy (C Daniel Batson et al., 1999; Lönnqvist et al., 2014), self-deception was found to be hardly diminished and quickly recovered even after repeatedly presenting the reality to people (Chance et al., 2015). Our previous study found that facilitating prosocial motives could effectively decrease deception in moral hypocrisy (Tang et al., 2017). Results in the current study support this finding by showing that prosocials were less deceptive and hypocritical than proselfs. Specifically, results in Experiment 2 suggest that enhancing the role of social goals such as social image concerns would also be effective to decrease moral hypocrisy, especially for proselfs. In addition, our findings also provide applications for treating individuals with different ways in deception prevention and reduction. For instance, measurements about individuals’ social orientation could be used firstly to identify individuals. Then, for individuals with prosocial orientations, emphasizing moral principles or cost for others in deception might be more useful in deception prevention and reduction than using cues related to social image management. However, for individuals with selfish orientations, highlighting the importance of others’ opinions or impression about their behaviors might be more effective.

One limitation of our research is that we did not directly manipulate factors related to self-image, making it hard to know how hypocritical fairness caused by self-deception would be changed. Self-deception has been used as a defense mechanism to serve “egoistic bias” and “moralistic bias” and maintain a positive perception of intellectual status and morality traits (Paulhus and John, 1998). When people perceive conflicts between moral standards and their actions for self-interest, they would use self-deception strategy to deal with these conflicts (Trivers, 1985; C Daniel Batson et al., 1999). Thus, in our study, participants wanted to obtain more but did not want to be directly truly unfair then they chose to be transparently fair even when they knew that their truth would be revealed. We did not find that self-deception motivated moral hypocrisy was changed with social context and evaluation from others, supporting the assumption that self-deception serves social advancement on the basis of self-enhancement rather than social image concerns in situations (Von Hippel and Trivers, 2011). Thus enhancing self-enhancement such as endorsing self-affirmation in behaviors or strengthening the power of moral standards by explicitly emphasizing them such as oath taking (Jacquemet et al., 2018), might be more useful in increasing or decreasing self-deception.

Besides, although we tried our best to control the effects of the lab settings or existence of an experimenter on participant by leaving them in a separate room or compartment to finish the task and telling them their totals would be only known by themselves before they made divisions, their deception was still affected by this context in Experiment 1 compared to the completely anonymous online context in Experiment 2. For proselfs, deception revelation decreased deception in Experiment 1 (R vs. UR: 57.03% vs. 85.36%), whereas this effect was weakened in Experiment 2 (R vs. UR: 68.38% vs. 78.07%); for prosocials, it did not affect deception in Experiment 1 (R vs. UR: 41.82% vs. 38.98%), but removing it increased deception in Experiment 2 (R vs. UR: 36.85% vs. 62.65%). These findings suggest that both prosocials and proselfs were sensitive to the lab context which might reveal their identity or ways of behaviors, and proselfs were more responsive to others’ evaluation compared to simple revelation in deception. Therefore, to increase the effects of being watched or observed on deception in social context (Lönnqvist et al., 2014), stronger manipulations about public claims such as adding both revelation and evaluation in deception prevention and reduction should be considered in future studies.

Overall, our findings contribute to understanding of the role of social value orientations and social image concerns in nonmaterial-motivated deception, moral hypocrisy. We hope that they can not only facilitate exploring the individual difference in deception, but also prove useful for future studies that aims to decrease deception and moral hypocrisy.

## Ethics Statement

This study was carried out in accordance with the recommendations of “Institutional Review Board of the State Key Laboratory of Cognitive Neuroscience and Learning at BNU” with written informed consent from all subjects. All subjects gave written informed consent in accordance with the Declaration of Helsinki. The protocol was approved by the Institutional Review Board of the State Key Laboratory of Cognitive Neuroscience and Learning at BNU.

## Author Contributions

HT developed the study concept. All authors contributed to the design. Testing and data collection were performed by ZL and SW. ZL and SW performed the data analysis and inpreparation of manuscript with HT and SS. WS-A, SS, and CL provided critical revisions. All authors approved the final version of the manuscript for submission.

## Conflict of Interest Statement

The authors declare that the research was conducted in the absence of any commercial or financial relationships that could be construed as a potential conflict of interest.
